# Development of a Validated UPLC-MS/MS Method for Analyzing Major Ginseng Saponins from Various Ginseng Species

**DOI:** 10.3390/molecules24224065

**Published:** 2019-11-09

**Authors:** Ling Yang, Chi-Lin Li, Yung-Yi Cheng, Tung-Hu Tsai

**Affiliations:** 1 Institute of Traditional Medicine, School of Medicine, National Yang-Ming University, Taipei 112, Taiwan; since199931208@gmail.com (L.Y.); a2233525@gmail.com (C.-L.L.); 2 Natural Products Research Laboratories, UNC Eshelman School of Pharmacy, University of North Carolina, Chapel Hill, NC 27599, USA; vininecheng@gmail.com; 3 Chinese Medicine Research and Development Center, China Medical University and Hospital, Taichung 40402, Taiwan; 4 Institute of Food Safety and Health Risk Assessment, National Yang-Ming University, Taipei 112, Taiwan; 5 Graduate Institute of Acupuncture Science, China Medical University, Taichung 40402, Taiwan; 6 Department of Chemical Engineering, National United University, Miaoli 36063, Taiwan

**Keywords:** ginseng species, ginsenosides, traditional Chinese medicine, ultra-performance liquid chromatography-tandem mass spectrometry

## Abstract

Ginsenosides, which contain one triterpene and one or more sugar moieties, are the major bioactive compounds of ginseng. The aim of this study was to develop and optimize a specific and reliable ultra-performance liquid chromatography-tandem mass spectrometry (UPLC-MS/MS) method for the analysis of twelve different resources of ginseng. The six marker compounds of ginsenoside Rb_1_, ginsenoside Rb_2_, ginsenoside Rc, ginsenoside Rd, ginsenoside Re, and ginsenoside Rg_1_, as well as an internal standard, were separated by a reversed-phase C-18 column with a gradient elution of water and methanol-acetonitrile. The multiple-reaction monitoring (MRM) mode was used to quantify and identify twelve market products. The results demonstrated that not only is the logarithm of its partition coefficient (cLog P; octanol-water partition coefficient) one of the factors, but also the number of sugars, position of sugars, and position of the hydroxyl groups are involved in the complicated separation factors for the analytes in the analytical system. If the amount of ginsenoside Rb_1_ was higher than 40 mg/g, then the species might be *Panax quinquefolius,* based on the results of the marker ginsenoside contents of various varieties. In summary, this study provides a rapid and precise analytical method for identifying the various ginsenosides from different species, geographic environments, and cultivation cultures.

## 1. Introduction

Ginseng has been used as a nutritional supplement and traditional Chinese medicine for centuries. Ginseng belongs to the Panax genus (Araliaceae family). When compared to other ginseng species, *Panax ginseng* C.A. Meyer, *Panax quinquefolius* L., and *Panax japonicus* C.A. Meyer are the most frequently used, and they are mainly known as Korean ginseng, American ginseng, and Japanese ginseng, respectively [1]. The first literature record of Shen-nung-ben-tsao-jing (The Holy Farmer’s Material Medica) ca. 25 A.D. cited ginseng as an imperial herb that was regarded as a vital energy supplement, a sedative [2], and an antifatigue agent [3], which had nontoxic characteristics and could be administered over the long term. The medicinal parts of ginseng are the root and rhizome. Many studies suggest that parts of leaves [4], radix, root hairs, and berries [5] possess antiaging and antioxidant effects. Therefore, ginseng is used as a dietary supplement in daily life.

Ginsenosides are the major active components of ginseng and they are generally regarded as standards for investigating the quality of ginseng herbs and complementary commercial products [6]. Several pharmacological properties of each ginsenoside, such as neuroprotective [7,8], anti-inflammatory [9], and anticarcinogenic activity [10], have been shown in past research. It has also been claimed that ginseng effectively treats all types of diabetes, slows biological aging [11], slows cardiovascular disease [12], and boosts the function of the immune system [13]. 

**Figure 1:**
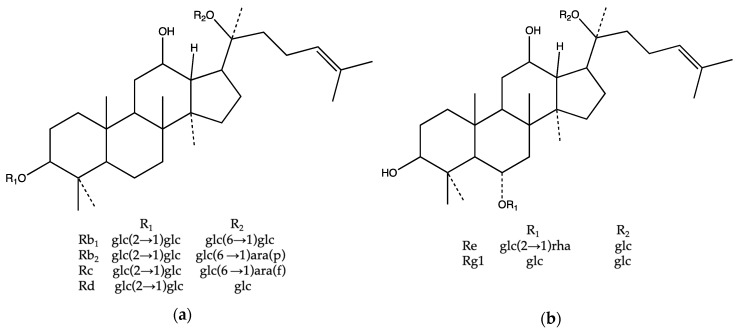
Structures of ginsenosides: (**a**) panaxadiol group and (**b**) panaxatriol group. The main ginsenosides used in this study are shown as follows: Glc, glucose; ara (p), arabinose in the pyranose form; ara (f), arabinose in the furanose form; and, rha, rhamnose.

The basic structure of ginsenoside is composed of a dammarane with four trans-rings of 17 carbon atoms. Ginsenosides are amphipathic, with five-carbon branched chains at C-20 and different numbers of sugar residues and hydroxyl (OH) groups at C-3, C-6, and C-20 [14]. To date, approximately 150 ginsenosides have been isolated and identified in the literature. Ginseng saponins can be divided into four structural groups: the panaxadiol group, the panaxatriol group, the ocotillol group, and the oleanolic acid group [15]. The panaxadiol and panaxatriol groups are structurally different at the sites of the sugar residues, and the panaxatriol moiety has a hydroxyl group at C-6. The panaxadiol group, including ginsenoside Rb_1_, ginsenoside Rb_2_, ginsenoside Rc, and ginsenoside Rd, and the panaxatriol group, including ginsenoside Rg_1_ and ginsenoside Re, contribute over 90% of the ginsenoside content of the ginseng genus [16] (Figure 1). Thus, in this study, these ginsenosides were selected as the standard for analyzing different ginseng resources. 

Previous studies demonstrated that many analytical approaches for the characterization of ginsenosides have been investigated, including thin layer chromatography (TLC), high-performance thin layer chromatography (HPTLC), high-performance liquid chromatography (HPLC), ultra-performance liquid chromatography (UPLC) with different detectors, gas chromatography (GC), and mass spectrometry (MS) [17,18]. The high molecular weights, chemical diversity, and structural similarity of these compounds have hindered simple and rapid ginsenoside analysis [19]. TLC was popular in ginseng research in the 1990s. HPTLC, which is more sensitive and accurate than using TLC plates, was used in the quantitative analysis of crude ginseng drugs [20]. HPLC was another favored method for ginsenosides and it was coupled with different detectors, including UV and photodiode array (DAD) [21]. Baseline interference resulted in a weak ginsenoside signal due to the poor absorption and unsuitable wavelengths in the range of 198 to 205 nm for UV detection [22]. Although the DAD provided multiple wavelength spectra, the sensitivity was lower than that of single spectrum UV. The use of GC was restricted by the characteristics of the compounds. GC was used to detect pesticides in ginseng samples since volatile compounds may not be present in the main ginsenosides [23]. Rapid analysis progress, and low consumption of the sample [24], especially mixtures of compounds (ginseng and its metabolites), a large proportion of ginseng analysis research is based on MS detection due to the high sensitivity [25]. MS is usually combined with HPLC or UPLC [26].

17 articles were found while surveying the keywords of ginseng, tandem mass spectrometry, and species on PubMed. Again, surveying the keywords of ginseng, tandem mass spectrometry, and cultivation environment on PubMed, two articles were found. Wang et al. (2016) demonstrated that the quality of ginseng roots and rhizomes that were collected from different areas of Jilin and Heilongjiang provinces of China was different due to growing environment, cultivation technology, etc. [19]. None of these publications discuss the use of the calculated partition coefficient (cLog P) for the correction of the retention time and ginseng analytes. 

Our hypothesis is that the sugar position in the chemical structure of ginsenoside might affect the retention time. To prove this hypothesis, the cLog P values were calculated by the ChemDraw Professional 16 system for the analytes. The aim of this study was to develop and optimize a specific and reliable ultra-performance liquid chromatography-tandem mass spectrometry (UPLC-MS/MS) method for the simultaneous determination of the major saponins in *P. ginseng*, *P. quinquefolius*, and *P. japonicus* ginseng since little is known about the correlation of the species or cultivation environment of ginseng to the ratio of six ginsenosides in the Asian and North American ginseng. The method was applied to determine the contents of ginsenoside Rb_1_, ginsenoside Rb_2_, ginsenoside Rc, ginsenoside Rd, ginsenoside Rg1, and ginsenoside Re in twelve different ginseng sources. 

## 2. Results and Discussion

### 2.1. Optimization of UPLC-MS/MS Conditions

**Figure 2:**
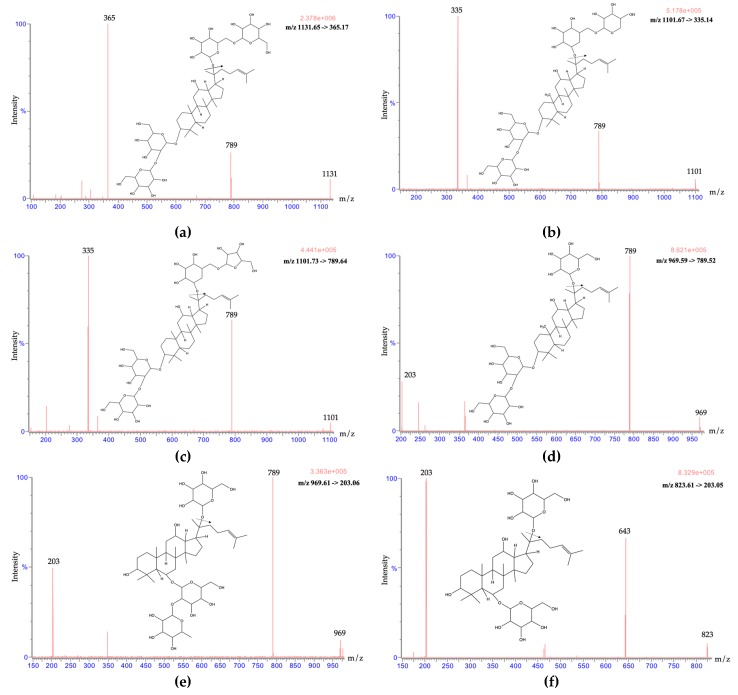
Product ion mass spectra of six marker compounds: (**a**) ginsenoside Rb_1_, (**b**) ginsenoside Rb_2_, (**c**) ginsenoside Rc, (**d**) ginsenoside Rd, (**e**) ginsenoside Re, and (**f**) ginsenoside Rg_1_.

**Figure 3:**
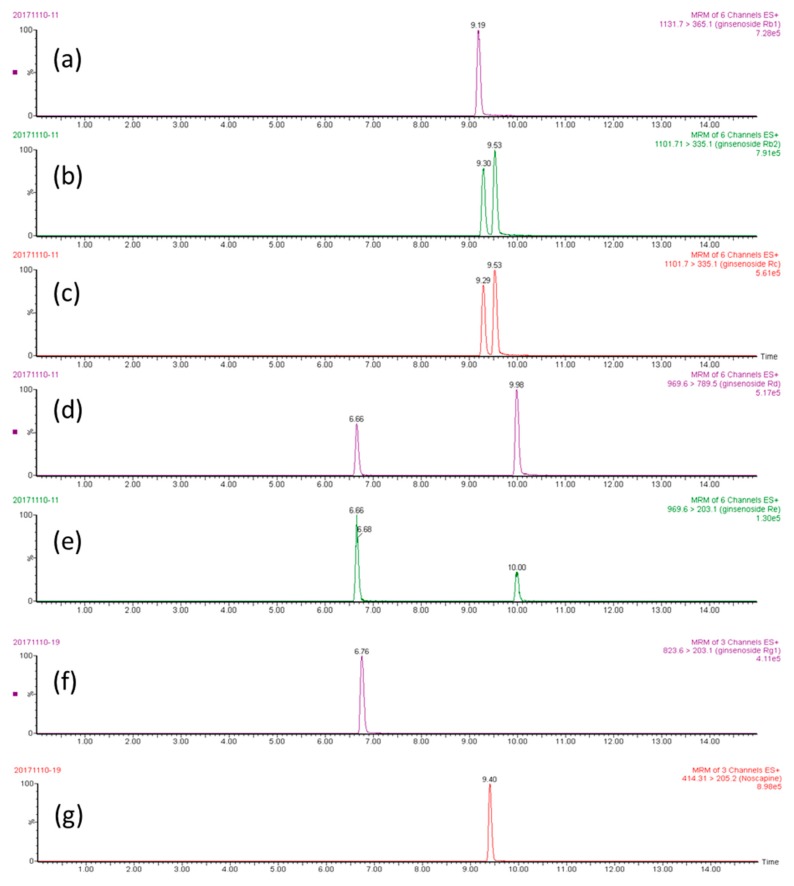
The multiple-reaction monitoring (MRM) chromatographs of analytes (500 ng/mL): (**a**) ginsenoside Rb_1_, (**b**) ginsenoside Rb_2_, (**c**) ginsenoside Rc, (**d**) ginsenoside Rd, (**e**) ginsenoside Re, (**f**) ginsenoside Rg_1_, and (**g**) noscapine (internal standard).

The stock solution (100 ng/mL) of ginsenoside Rb_1_, ginsenoside Rb_2_, ginsenoside Rc, ginsenoside Rd, ginsenoside Re, ginsenoside Rg_1_, and noscapine was used to optimize the UPLC-MS/MS conditions. Both positive and negative electrospray were optimized at first. In the tuning file of the Waters Acquity UPLC^TM^ system, the ion fragments under positive ESI conditions were more stable and display higher intensities when compared with the negative mode. Thus, a positive electrospray was applied for the following analyte identification (Figure 2). The multiple-reaction monitoring (MRM) mode could not only detect multiple compounds in a single run, but also appeared to be highly selective for the quantification of twelve market products and their identification. The chromatographs showed high intensity, high sensitivity, and clear peak shapes. To optimize the separation of analytes, while using methanol alone, the peak shapes were not sharp enough, and a peak tailing phenomenon appeared. Therefore, a mixed organic solvent of methanol and acetonitrile at a volume ratio of 4:1 produced the best peak shape and it was selected as the organic phase (Figure 3). 

Regarding the mass spectrometry conditions, the capillary voltage was downregulated from 4.0 kV to 3.8 kV, which acquired a steady high intensity. For the cone voltage, although each ginsenoside has a best cone voltage condition, in the Waters Acquity UPLC^TM^ system, the same cone voltage had to be set for all ginsenosides. The optimized cone voltage that was provided by the Waters Acquity UPLC^TM^ system was 40 V for all ginsenosides. We checked the cone voltages from ± 5 V (35 V–45 V), eventually using 40 V for the ginsenosides. The analytes were quantified in the MRM mode at *m*/*z* 1131.65, 365.14 for ginsenoside Rb_1_; *m*/*z* 1101.67, 335.13 for ginsenoside Rb_2_; *m*/*z* 1101.73, 335.14 for ginsenoside Rc; *m*/*z* 969.59, 789.52 for ginsenoside Rd; *m*/*z* 969.01, 789.52 for ginsenoside Re; *m*/*z* 823.61, 203.05 for ginsenoside Rg_1_; and, *m*/*z* 645.54 and *m*/*z* 414.29, 220.11 for noscapine.

Several studies comparing Asian and American ginseng ingredients via UPLC-MS have already been published; however, UPLC-MS/MS provides a higher level of sensitivity and specificity than UPLC-MS due to UPLC-MS/MS detecting signals through both parent ions and product ions, while UPLC-MS only detects the parent ions. For the analysis time, analyzing the fractions is time consuming and the elution time was 40 minutes for a single run. Furthermore, the ginsenosides of main concern were quite different [27]. Another two-dimensional LC-MS method was published for the global profiling of ginsenosides. The article also mentioned that the PDA detector was set between 190, 203, and 400 nm, which might cause inaccuracies in the quantification due to the signals at wavelengths below 220 nm [28]. Electrospray ionization (ESI) and atmospheric pressure chemical ionization, which evaporates the solvent and sample from a gas into ions, are generally used [29]. A multistage LC-MS/MS, which provides a high level of sensitivity and specificity, was chosen for use in this study. As shown, ginseng analytical methods are commonly time consuming [30]. When compared with previous reports, the advantage of this study is that focuses on the six main ginsenosides (Rb_1_, Rb_2_, Rc, Re, Rd, and Rg_1_), which account for 90% of the contents of the three main ginseng species. The single analysis duration was only 12 minutes, and the relationship between the structure and the retention time is discussed for different ginseng sources.

### 2.2. Method Validation

The calibration curves showed good linearity in the range of 10–500 ng/mL for all ginsenosides. The calibration curves and correlation coefficients (r^2^) were as follows: y = 0.0012x − 0.001 (r^2^ = 0.999, ginsenoside Rb_1_), y = 0.0013x + 0.0034 (r^2^ = 0.999, ginsenoside Rb_2_), y = 0.001x − 0.0027 (r^2^ = 0.999, ginsenoside Rc), y = 0.0008x − 0.0021 (r^2^ = 0.999, ginsenoside Rd), y = 0.0002x − 0.0002 (r^2^ = 0.999, ginsenoside Re), and y = 0.0004x + 0.009 (r^2^ = 0.999, ginsenoside Rg_1_). The accuracy and precision were evaluated by intraday and interday assays. The RSD and accuracy were calculated to be within the range of 1.38%–6.68% and (−)8.86%–12.88% for intraday assays (Table 1), respectively, which correspond to the U.S. Food and Drug Administration bioanalytical method validation guidance. The lower limit of detection (LOD) is 10 ng/mL and lower limit of quantification (LOQ) is 25 ng/mL, which were calculated by diluting the standard solution to when the signal-to-noise ratios (S/N) of the analytes were approximately 3 and 10, respectively [31].

### 2.3. The Correlation between the Chemical Structure and Retention Time

cLog P was applied to evaluate the correlation between the retention time and the analytes to investigate the correlation between the chemical structure and separation. A lower cLog P value might represent a higher polarity, which might cause ginsenoside Rb_1_ to be eluted before ginsenoside Rb_2_. Thus, the cLog P was applied to evaluate the correlation of the retention time and the ginseng analytes. 

The differences in the chemical structures between the panaxatriol and panaxadiol ginsenosides and the basic dammarane are the position of the sugar on C-6 and the number of hydroxyl (OH) groups on C-3. The panaxatriol group is more polar than the panaxadiol group due to the additional hydroxyl (OH) group on C-3. Thus, ginsenoside Re and ginsenoside Rg_1_ have shorter retention times than the others. Comparing ginsenoside Rb_1_ and ginsenoside Rb_2_, the functional groups on the R2 site were glucose and arabinose, respectively, which caused a lower cLog P value for ginsenoside Rb_1_. The lower cLog P value represented a higher polarity, because ginsenoside Rb_1_ eluted before ginsenoside Rb_2_. Ginsenoside Rb_2_ (pyranose form at the R2 site) and ginsenoside Rc (furanose form) are isomers, and their retention times can also be explained by cLog P. While the compounds have similar structures, the form of arabinose was not the same. Ginsenoside Rd possesses less sugar residues at C-20 when compared with other panaxadiol group ginsenosides; therefore, it is less polar than other ginsenosides and it has the longest elution time (Table 2). The results demonstrated that cLog P is not the sole factor, and the number of sugars, position of the sugars, and position of the hydroxyl groups were involved in the complicated separation factors for the analytes in the analytical system. These results are consistent with a previous report [32], showing that the partition coefficient is one of the factors that correlates with the retention time as well as the rest of the factors, such as the number of functional groups or the position of the functional groups.

### 2.4. Ginseng Sample Quality

According to Table 3, each species contained a consistent amount of each ginsenoside compound. When compared to the ginseng sample C (Korean ginseng) and ginseng sample F (Japanese ginseng), *P. quinquefolius* (sample G) had the highest content of ginsenoside Rb_1_, ginsenoside Rd, and ginsenoside Re, but the lowest amount of ginsenoside Rb_2_ and ginsenoside Rg_1_. *P. ginseng* had the highest concentrations of ginsenoside Rb_2_ (around the average of 3.78 mg/g) and ginsenoside Rg_1_ (average of 6.4 mg/g), which was the opposite result to that found in *P. quinquefolius* L. Our results are in agreement with those of the Chen et al report, where ginsenoside Rb_2_ and ginsenoside Rc were more likely to be present in Asian ginsengs, whereas ginsenoside Rb_1_ and ginsenoside Rd tended to be present in American ginsengs [33]. 

**Figure 4:**
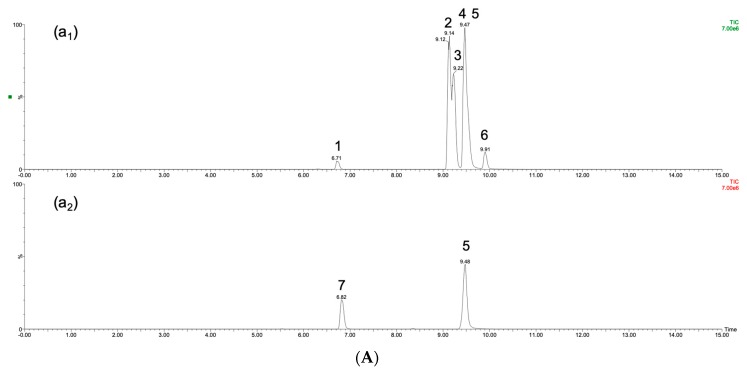

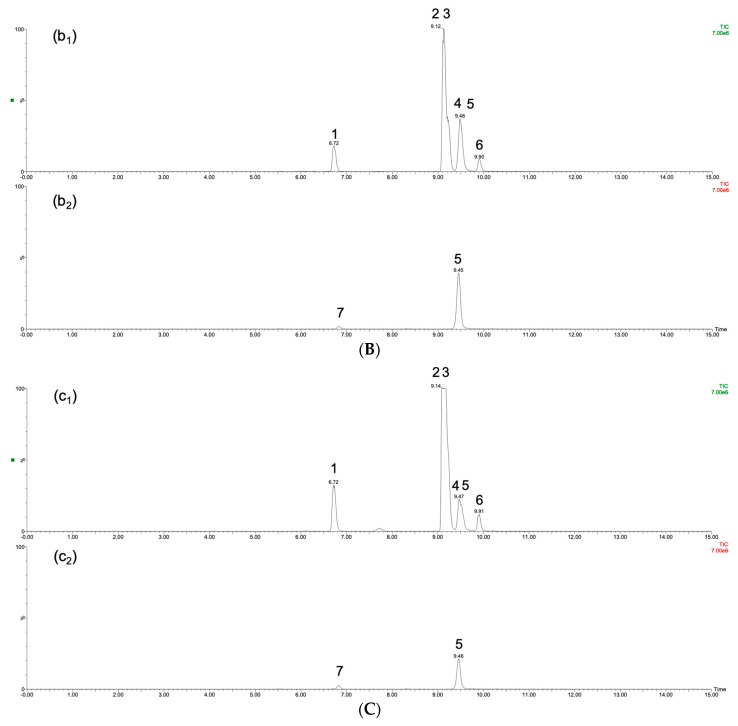
The TIC chromatographs of (**A**) The sample B of *P. ginseng*; (**B**) The sample F of *P. japonicus*; (**C**) The sample J of *P. quinquefolius*; peak (1) ginsenoside Re (2) ginsenoside Rb_1_ (3) ginsenoside Rb_2_ (4) ginsenoside Rc (5) noscapine (internal standard) (6) ginsenoside Rd (7) ginsenoside Rg_1_.

*P. japonicus* had the lowest contents of ginsenoside Rb_1_ and ginsenoside Rd, and ginsenoside Re was undetectable in sample E and sample F. The compositions of the ginsenosides in *P. ginseng* and *P. japonicus* were more similar, but Japanese ginseng (sample E and sample F) had obviously low concentrations of ginsenoside Rd. Figure 4 provides the three ginseng species profiles, including samples A, F, and J, representing *P. ginseng, P. japonicus,* and *P. quinquefolius,* respectively. Figure 4 indicates that the content of ginsenoside Rb_1_ in *P. quinquefolius* was far above that in other species, and it was difficult to detect ginsenosides Re and Rg_1_ in *P. japonicus*. Table 3 also shows that the contents of ginsenosides in sample I were far below those in the other American ginseng samples, and these differences might be attributed to the cultivation environment. Wild-simulated samples displayed the difficulties that are typically shown by traditional Chinese medicines regarding consistent quality. The method that is provided in this study will be a good approach for evaluating the quality of ginseng. Different culturing areas may provide distinct soil conditions, temperate climate, humidity, and even altitude, all of which will affect the contents of ginsenosides.

In general, five geographical species provide the total ginseng supply: South Korea (*P. ginseng*), China (*P. ginseng*), Japan (*P. japonicus*), Canada (*P. quinquefolius*), and the United States (*P. quinquefolius*) [1]. For high-quality ginseng production, ginseng requires a cool and temperate climate and special soil conditions, including soil that is nutrient rich, slightly acidic, well drained, and under deep shade [34]. Three different ginseng cultivation methods were analyzed in this study: truly wild, wild simulated, and wood cultivated. In truly wild cultivation, the plants were free to grow without any management by humans. In wild-simulated cultivation, the natural conditions of temperature and humidity for ginseng growth were replicated, but the soil conditions could not meet the requirements. In the wood-cultivated method, ginseng was grown with intensive field plowing and artificial shade structures. Except during the winter, the ginseng beds were protected and covered with floating plastic to create shade, strengthen photoselectivity, and protect against heavy rains [35]. 

In the study by Chen et al (2019), the average contents of ginsenosides indicated that the distinct volcanic pumice soil conditions of New Zealand might be more suitable for ginseng cultivation than original native locations (China and Korea) [36]. Different ginseng processing methods and cultivation environments [37] may be required due to variations in the amounts of ginsenosides [38]. *P. ginseng* (Korean ginseng), which is mainly known as red ginseng and white ginseng, has a distinct preparation method. A steaming and drying process is used to produce red ginseng. The steaming step might elevate the contents of ginsenosides in the panaxadiol and panaxatriol groups [39]. American ginseng is mostly cultivated in Canada and the northern United States. However, due to a supply shortage and price fluctuations, American ginseng has recently been cultivated in northern China. The contents of the ginsenosides might change accordingly. Therefore, the relative proportions of ginsenosides might indicate the market type of ginseng. 

## 3. Materials and Methods

### 3.1. Chemicals and Reagents

The chemical standards of ginsenoside Rb_1_, ginsenoside Rb_2_, ginsenoside Rc, ginsenoside Rd, ginsenoside Re, and ginsenoside Rg_1_ were purchased from Sigma-Aldrich (St. Louis, MO, USA). LC-MS grade solvents, including acetonitrile and methanol, were procured from J.T. Baker, Inc. (Phillipsburg, NJ, USA). Triple deionized water (Millipore Bedford, MA, USA) was prepared for all aqueous solutions.

### 3.2. Origin of Ginseng Samples

Twelve different market ginseng samples were analyzed in this experiment (Table 3). Four different *P. ginseng* sources were obtained and two *P. japonicus* were analyzed. All of the market *P. ginseng* and *P. japonicus* were wood cultivated. Six *P. quinquefolius* samples were provided for this experiment, including truly wild, wood-cultivated, and wild-simulated market ginseng. Dr. Wen-Ya Peng identified the origins of ginseng material samples according to her medical doctor specialty.

### 3.3. Preparation of Standard Solutions

Standard stock solutions of ginsenoside Rb_1_, ginsenoside Rb_2_, ginsenoside Rc, ginsenoside Rd, ginsenoside Re, ginsenoside Rg_1_, and noscapine were prepared at a concentration of 1 mg/mL in methanol. All of the stock solutions were stored at −20 °C before use.

### 3.4. Preparation of Ginseng Samples

The ginseng (root part) was cut into pieces and weighed; then, the ginseng slice (1 g) was soaked in a tube with 6 mL of 50% ethanol for 30 min. The tube was then incubated and sonicated at 37 °C for 6 h. The samples were filtered through a 0.22 μm Millipore filter. A rotary evaporator was used to ensure that the ethanol residue was eliminated. Subsequently, the sample was freeze-dried to obtain a powder.

The ginseng-extracted powder was added to 50% methanol to obtain a 1 mg/mL concentration, centrifuged at 13,000 rpm for 5 min. (ThermoFisher FRESCO 17, Dreieich, Germany), and then filtered through a 0.22 μm Millipore filter. To meet the range of calibration curves, all the samples were diluted 50-100X with 50% methanol before analysis. All analytes were prepared as above before injection into the UPLC-MS/MS for analysis.

### 3.5. Instruments and Conditions

The UPLC-MS/MS system consisted of a Waters Acquity UPLC^TM^ system (Waters Co., Milford, MA, USA), a binary solvent manager, an automatic liquid chromatographic sampler, and a Waters Xevo^TM^ tandem quadrupole mass spectrometer equipped with an ESI source. All ion transitions and collision energies were determined and optimized while using the MassLynx 4.1 software data platform (Waters). The mass spectrometry conditions were set, as follows: ESI, positive mode; desolvation temperature, 550 °C; collision gas, argon; nebulizing gas, nitrogen; source temperature, 150 °C; desolvation gas flow, 800 L/h; capillary voltage, 3.8 kV; and, cone gas flow, 60 L/h. The optimized cone voltage was 40 V for all of the ginsenosides. The ion transitions monitored were *m/z* 1131.65, 365.17 for ginsenoside Rb_1_; *m/z* 1101.67, 335.14 for ginsenoside Rb_2_; *m/z* 1101.73, 789.64 for ginsenoside Rc; *m/z* 969.59, 789.52 for ginsenoside Rd; *m/z* 969.61, 203.06 for ginsenoside Re; *m/z* 823.61, 203.05 for ginsenoside Rg_1_; and, *m/z* 414.29, 220.11 for noscapine (Figure 2). Noscapine was used as the internal standard (IS) for these analytes. The analytical column for ginsenoside separation was a Purospher^®^ STAR RP-18 end-capped column (100 × 2.1 mm, 2 μm, Merck KGaA, Darmstadt, Germany) maintained at a temperature of 40 °C in the column oven. Mobile phase A consisted of triple-distilled water and mobile phase B consisted of acetonitrile: methanol 1:4 (*v*/*v*). The mobile phase was filtered through a 0.22 μm filter and then degassed by a sonicator for one hour before use. The gradient elution was 80% to 5% A in 0.01–10 minutes and back to 80% A in 10–15 minutes. The flow rate was 0.2 mL/min., and the injection volume was 10 μL. The MassLynx 4.1 software (Waters Corporation, Milford, MA, USA) data platform was used for spectral acquisition, spectral presentation, and peak quantification.

### 3.6. Validation of the Analytical Method

The method was validated while using current U.S. Food and Drug Administration bioanalytical method validation guidance. The accuracy, precision, and calibration curves were evaluated. The intraday variability was determined by quantifying six repetition sets of different concentrations on the same day. The accuracy (bias) was calculated from the nominal concentration (C_nom_) and the mean value of observed concentrations (C_obs_) while using the following formula: Bias (%) = [(C_obs_ − C_nom_)/C_nom_] × 100. The precision, as the relative standard deviation (RSD), was calculated, as follows: RSD (%) = [standard deviation (S.D.)/C_obs_] × 100. All of the linear calibration curves were required to have a coefficient of estimation of at least 0.995.

## 4. Conclusions

This study provided methods for evaluating the quality of the traditional Chinese medicine ginseng. A rapid and validated UPLC-MS/MS detection system was developed for the simultaneous determination of ginsenoside Rb_1_, ginsenoside Rb_2_, ginsenoside Rc, ginsenoside Rd, ginsenoside Re, and ginsenoside Rg_1_. The method was successfully applied to the analysis of twelve different ginseng sources. The results of this study can be used to determine the tendencies of the ginseng species. 

## Figures and Tables

**Table 1:** Method validation of the intraday precision and accuracy for the ten determinations of ginsenosides from standard samples.

Compound	Nominal Concentration (ng/mL)	Intraday		
		Observed Concentration (ng/mL)	Precision RSD (%)	Accuracy Bias (%)
ginsenoside Rb_1_	25	25.93 ± 0.94	3.61	3.70
	250	275.2 ± 3.80	1.38	10.06
	500	500.7 ± 31.42	6.27	0.13
ginsenoside Rb_2_	25	23.79 ± 0.82	3.43	−4.86
	250	278.8 ± 6.38	2.29	11.54
	500	458.8 ± 29.00	6.32	−8.52
ginsenoside Rc	25	26.37 ± 0.61	2.33	5.49
	250	250.5 ± 5.6	2.23	0.19
	500	464.5 ± 31.02	6.68	−7.11
ginsenoside Rd	25	28.21 ± 0.82	2.90	12.5
	250	266.9 ± 9.38	3.52	6.76
	500	480.3 ± 23.44	4.88	−3.94
ginsenoside Re	25	28.22 ± 0.70	2.50	12.88
	250	277.6 ± 9.19	3.31	11.06
	500	533.2 ± 11.59	2.17	6.64
ginsenoside Rg_1_	25	22.78 ± 0.59	2.58	−8.86
	250	259.1 ± 3.87	1.49	3.65
	500	489.3 ± 15.77	3.22	−2.14

Data were expressed as the mean ± SD (n = 6).

**Table 2:** Chemical structure and retention time of each ginsenoside. The cLog P values were calculated by the ChemDraw program 16 system.

Ginsenoside	Structure Type	Molecular Formula	cLog P Value	No. of Sugars	Position of Sugar			Position of OH		Retention Time
					C-3	C-6	C-20	C-3	C-12	
Re	panaxatriol	C_48_H_82_O_18_	2.18	3	0	2	1	1	1	6.75
Rg_1_	panaxatriol	C_42_H_72_O_14_	2.27	2	0	1	1	1	1	6.85
Rb_1_	panaxadiol	C_54_H_92_O_23_	1.64	4	2	0	2	0	1	9.21
Rb_2_	panaxadiol	C_53_H_90_O_22_	1.67	4	2	0	2	0	1	9.32
Rc	panaxadiol	C_53_H_90_O_22_	2.29	4	2	0	2	0	1	9.55
Rd	panaxadiol	C_48_H_82_O_18_	1.63	3	2	0	1	0	1	10.01

**Table 3:** Twelve different sources of ginseng were provided for this study. Contents of ginsenoside Rb_1_, ginsenoside Rb_2_, ginsenoside Rc, ginsenoside Rd, ginsenoside Re, and ginsenoside Rg_1_ of the twelve ginseng sources. The average content of ginsenosides is categorized by the scientific name and cultivation method.

Scientific Name (Common Name)	Cultivation Region	Cultivation Method	Sample No.	Rb_1_ (mg/g)	Rb_2_ (mg/g)	Rc (mg/g)	Rd (mg/g)	Re (mg/g)	Rg_1_ (mg/g)
*P. ginseng* C.A. Meyer (Korean ginseng)	Korea	Wood- cultivated	A	10 ± 1	5 ± 1	5 ± 1	3 ± 1	1 ± 0.3	7 ± 1
			B	6 ± 1	2 ± 0.4	2 ± 0.3	2 ± 0.2	ND	5 ± 1
			C	16 ± 0.4	4 ± 0.1	3 ± 0.1	5 ± 0.1	1 ± 0.0	9 ± 1
			D	9 ± 1	4 ± 0.4	4 ± 1	3 ± 1	0.1 ± 0.1	5 ± 0.4
			Mean	10 ± 4 *	4 ± 1 *	3 ± 1	3 ± 1 *	0.5 ± 0.4 *	6 ± 2
*P. japonicus* C.A. Meyer (Japanese ginseng)	Japan	Wood- cultivated	E	7 ± 0.4	4 ± 0.2	3 ± 0.2	3 ± 0.3	ND	5 ± 1
			F	3 ± 1	4 ± 1	ND	2 ± 0.4	ND	ND
			Mean	5 ± 2 *	4 ± 0.1 *	2 ± 0.0	2 ± 1 *	ND	3 ± 0.4
*P. quinquefolius* L. (American ginseng)	Canada	Truly wild	G	42 ± 1	1 ± 0.1	5 ± 0.1	43 ± 1	3 ± 0.2	7 ± 0.1
	United States		H	41 ± 3	1 ± 0.1	5 ± 0.3	22 ± 1	9 ± 1	1 ± 0.1
	Wisconsin		I	39 ± 2	0.1 ± 0.0	2 ± 0.2	31 ± 2	6 ± 1	3 ± 0.2
			Mean	41 ± 1 *	1 ± 0.3 *	4 ± 2	32 ± 10 *	6 ± 3 *	3 ± 0.1
		Wood- cultivated	J	51 ± 4	1 ± 0.1	5 ± 1	26 ± 2	3 ± 1	1 ± 0.1
			K	46 ± 4	0.4 ± 0.1	3 ± 1	15 ± 2	3 ± 1	12 ± 4
			Mean	48 ± 3 *	1 ± 0.1 *	4 ± 2	20 ± 7 *	3 ± 0.2 *	6 ± 8
		Wild- simulated	L	4 ± 0.4	0.7 ± 0.1	1 ± 0.1	1 ± 0.2	ND	6 ± 1

* *p* < 0.05; Means within a scientific name and each ginsenoside are significantly different as calculated by the ANOVA test in SPSS software. Data were expressed as the mean ± SD. (n = 3); ND: not detectable.
